# Deltamethrin and transfluthrin select for distinct transcriptomic responses in the malaria vector *Anopheles gambiae*

**DOI:** 10.1186/s12936-023-04673-5

**Published:** 2023-09-04

**Authors:** Marius Gonse Zoh, Jean-Marc Bonneville, Frederic Laporte, Jordan Tutagata, Christabelle G. Sadia, Behi K. Fodjo, Chouaibou S. Mouhamadou, Justin McBeath, Frederic Schmitt, Sebastian Horstmann, Stéphane Reynaud, Jean-Philippe David

**Affiliations:** 1 Laboratoire d’Ecologie Alpine (LECA), Grenoble-Alpes University, Savoie Mont-Blanc University, CNRS, 38041 Grenoble, France; 2 Vector Control Product Evaluation Centre (VCPEC) Institut Pierre Richet (VCPEC IPR)/INSP, Bouaké, Côte d’Ivoire; 3 Centre Suisse de Recherches Scientifiques, Abidjan, Côte d’Ivoire; 4 Envu, Milton Hall, Ely Road. Milton, Cambridge, CB24 6WZ UK; 5 Envu, 2022 Environmental Science FR S.A.S, 3 Place Giovanni Da Verrazzano, 69009 Lyon, France; 6 Envu, 2022 ES Deutschland GmbH, Alfred-Nobel-Straße 50, 40789 Monheim, Germany

**Keywords:** *An. gambiae*, Transfluthrin, Deltamethrin, Metabolic resistance, Transcriptomics

## Abstract

**Background:**

The widespread use of pyrethroid insecticides in Africa has led to the development of strong resistance in *Anopheles* mosquitoes. Introducing new active ingredients can contribute to overcome this phenomenon and ensure the effectiveness of vector control strategies. Transfluthrin is a polyfluorinated pyrethroid whose structural conformation was thought to prevent its metabolism by cytochrome P450 monooxygenases in malaria vectors, thus representing a potential alternative for managing P450-mediated resistance occurring in the field. In this study, a controlled selection was used to compare the dynamics of resistance between transfluthrin and the widely used pyrethroid deltamethrin in the mosquito *Anopheles gambiae*. Then, the associated molecular mechanisms were investigated using target-site mutation genotyping and RNA-seq.

**Methods:**

A field-derived line of *An. gambiae* carrying resistance alleles at low frequencies was used as starting material for a controlled selection experiment. Adult females were selected across 33 generations with deltamethrin or transfluthrin, resulting in three distinct lines: the Delta-R line (selected with deltamethrin), the Transflu-R line (selected with transfluthrin) and the Tiassale-S line (maintained without selection). Deltamethrin and transfluthrin resistance levels were monitored in each selected line throughout the selection process, as well as the frequency of the L1014F *kdr* mutation. At generation 17, cross-resistance to other public health insecticides was investigated and transcriptomes were sequenced to compare gene transcription variations and polymorphisms associated with adaptation to each insecticide.

**Results:**

A rapid increase in resistance to deltamethrin and transfluthrin was observed throughout the selection process in each selected line in association with an increased frequency of the L1014F *kdr* mutation. Transcriptomic data support a broader response to transfluthrin selection as compared to deltamethrin selection. For instance, multiple detoxification enzymes and cuticle proteins were specifically over-transcribed in the Transflu-R line including the known pyrethroid metabolizers *CYP6M2*, *CYP9K1* and *CYP6AA1* together with other genes previously associated with resistance in *An. gambiae*.

**Conclusion:**

This study confirms that recurrent exposure of adult mosquitoes to pyrethroids in a public health context can rapidly select for various resistance mechanisms. In particular, it indicates that in addition to target site mutations, the polyfluorinated pyrethroid transfluthrin can select for a broad metabolic response, which includes some P450s previously associated to resistance to classical pyrethroids. This unexpected finding highlights the need for an in-depth study on the adaptive response of mosquitoes to newly introduced active ingredients in order to effectively guide and support decision-making programmes in malaria control.

**Supplementary Information:**

The online version contains supplementary material available at 10.1186/s12936-023-04673-5.

## Background

Insecticide-treated nets (ITNs) and indoor residual spraying (IRS) have contributed to reducing the number of malaria cases over the past decades in malaria-endemic countries [1]. Four classes of insecticides are authorized for ITNs and IRS: pyrethroids, carbamates, organophosphates, and organochlorines. However, only pyrethroids are recommended for ITNs [2]. Pyrethroids are neurotoxic insecticides that target the voltage-gated sodium channel, causing prolonged depolarization of neurons with repetitive discharges and synaptic disruption leading to paralysis and death of the insect [3]. Class I and Class II pyrethroids are mainly used for ITNs. They both have a phenoxybenzyl group as alcohol moiety, with type I pyrethroids, such as permethrin, lacking a cyano group in the α-position as opposed to type II pyrethroids, such as deltamethrin. Transfluthrin belongs to another pyrethroid category with an acidic moiety similar to Class I and class II pyrethroids, but with a polyfluorinated benzyl ring as alcohol moiety. Polyfluorobenzyl pyrethroids are semi-volatile and mainly used as household insecticides in different parts of the world [4].

To date, the extensive use of pyrethroids in public health as well as in agriculture has led to the selection and spread of resistance in major malaria vectors, threatening the effectiveness of vector control interventions [5, 6]. Insecticide resistance can be the consequence of a variety of physiological changes in insects, including mutation of the proteins targeted by insecticides (target site insensitivity), reduced insecticide penetration (cuticle thickening/modification) and biodegradation/sequestration of insecticides by detoxification enzymes (metabolic resistance) [7]. Target site insensitivity and metabolic resistance are the best-known pyrethroid resistance mechanisms in mosquitoes, although cuticle thickening and other mechanisms can also contribute to the overall phenotype. Target site insensitivity involves non-synonymous mutations affecting the voltage-gated sodium channel targeted by pyrethroids (VGSC gene, *kdr* mutations) [8]. Two distinct mutations occur in the S6 transmembrane segment of VGSC domain II at the L1014 locus (L995 using *Anopheles gambiae* VGSC codon nomenclature [9]). The *kdr* west mutation (L1014F) spread from west Africa while the *kdr* east mutation (L1014S) was first identified in east Africa [10, 11]. In addition, another mutation between domains III and IV N1575Y (N1570Y using *An. gambiae* VGSC codon nomenclature [9]) has also been described in *An. gambiae* populations in west Africa [12]. This mutation is associated with the L1014F mutation and is thought to compensate for its deleterious effects and/or to synergize its effect through allosteric interactions [13, 14]. As opposed to *An. gambiae* and *Anopheles coluzzii*, *kdr* mutations have not been described in *Anopheles funestus* suggesting a key role of metabolic resistance mechanisms in this species. Metabolic resistance results in an increased biodegradation of insecticide molecules before they reach their nervous target. This resistance mechanism is common across insects and mainly involves four detoxification enzymes families: cytochrome P450 monooxygenases (P450 or *cyp* genes), carboxy/cholinesterases (CCEs), glutathione S-transferases (GSTs) and UDP glucosyl transferases (UGTs) [15–18]. In addition, it has also been shown that other enzyme families, transporters and insecticide-binding proteins can contribute to the resistance phenotype [19–21]. In Africa, P450s are most associated with pyrethroid resistance in malaria vectors including *An. gambiae* and *An. funestus* [13, 22–24]. P450-mediated metabolism of type I and type II pyrethroids is thought to primarily start by the oxidation of the phenoxybenzyl ring of the alcohol side chain [25]. In contrast, transfluthrin might not be subjected to such oxidation as it contains a different alcohol side chain with a tetrafluorobenzyl ring. This idea is indeed backed by recent studies where several P450 inhibitors, including piperonyl butoxide (PBO), failed to restore full or partial susceptibility to polyfluorobenzylated pyrethroids in resistant mosquitoes showing elevated P450 activity [26, 27]. Although these studies support transfluthrin as being less prone to be metabolized by the P450s commonly associated with resistance to type I and type II pyrethroids, its potential to select for novel or existing resistance alleles is still unclear though of major importance for guiding the choice of alternative insecticides to be used in public health.

In this context, the aim of the study was to perform a multigenerational selection experiment for comparing the potential of a polyfluorinated pyrethroid (transfluthrin) and a type II phenoxybenzyl pyrethroid (deltamethrin) to select for resistance in the mosquito *An. gambiae* and to investigate the associated resistance mechanisms. This was achieved by selecting a field-derived *An. gambiae* line carrying pyrethroid resistance alleles at low frequencies for 33 generations with deltamethrin or transfluthrin. Resistance levels were monitored in each selected line and resistance mechanisms were studied by genotyping *kdr* mutations and RNA-seq.

## Methods

### Mosquitoes

A field-derived colony of *An. gambiae *sensu stricto (*s.s.*) (Tiassalé-S line) obtained from a controlled cross between the Tiassalé multi-resistant line originating from southern Côte d'Ivoire and the fully susceptible line Kisumu [28] was used as the parental line in this study. Larvae were reared in deionized water and fed with TetraMin® fish flakes. Adults were fed on filter papers impregnated with a 5% honey solution and blood feeding of adult females was performed on mice. All the lines described thereafter were maintained in the LECA tropical insectaries under the following conditions: 28 °C, 90% relative humidity, 14 h/10 h light/dark.

### Controlled selection

The Tiassalé-S line was equally divided into three lines. The first line (Tiassalé-S) was kept without selection and used as control. The other two lines were selected in parallel with deltamethrin (Delta-R line) or transfluthrin (Transflu-R line). Insecticide selections were made using 250 ml glass bottles coated with insecticides obtained as pure active ingredients from Sigma and diluted in 100% acetone. For each line, at least 15 batches of 20 non-blood-fed adult females were exposed to insecticide in bottles coated with 1 mL of insecticide solution. The doses used for selection were initially calibrated to achieve 50–70% mortality in the Tiassalé-S line as follows: deltamethrin 10 μg/mL and transfluthrin 0.25 μg/mL with exposure times of 15 min and 30 min respectively. Exposure times were slightly adjusted during the selection process in order to maintain 50 to 70% mortality. Mortality rates were recorded after a 24-h recovery time and survivors were blood fed and allowed to lay eggs for the next generation. Selection was performed for each line at each generation up to generation G33, except for generations G3, G4, G8, G13, G15, G17, G20, G22, G27, G29, G32 for which population size was considered as too low for at least one line (< 200 emerging females).

### Resistance monitoring

All bioassays were performed using non-blood fed, 3–5 days old females and at least 5 batches of 20 females per line. Insecticide exposure time was fixed at 1 h and mortality rate was recorded after a 24-h recovery time. The resistance monitoring of each line to its respective insecticide (deltamethrin and transfluthrin) was carried out at generations G0 (parental line), G2, G5, G7, G9, G11, G13, G17 and G33 using CDC glass bottles coated with the same dose as initially used for selection at G0. The resistance level of the Tiassale-S line to each insecticide was also monitored at the same generations.

Cross-resistance to other vector control insecticides was investigated at G17 for each selected line as compared to the control line. Bioassays were performed, using WHO test tubes equipped with impregnated filter papers following WHO standard procedures [29]. Insecticides tested were fenitrothion (1%), bendiocarb (0.5%) and DDT (4%). Mortality differences between each selected line and the control line Tiassalé-S were tested using Fisher's exact tests on mortality proportions.

### kdr mutation genotyping

The *kdr* west *L1014F* mutation was identified in the Tiassalé-S line prior to the start of the selection experiment and was then monitored for each selected line by individual genotyping at generations G0, G2, G7, G9 and G17. The *kdr* east L1014S mutation was not initially present in the Tiassale-S line and, therefore, not genotyped during the selection process. A total of 30 adult females non-exposed to insecticides were used for genomic DNA extraction by the cetyl trimetylammonium bromide (CTAB) method [30]. Genomic DNA was suspended in 20 µL of nuclease-free water and quantified using the Qubit DNA BR assay (Thermofisher Scientific) before dilution to 0.5 ng/µL. The *kdr* genotyping was performed using the allele-specific TaqMan qPCR method described in Bass and collaborators [31]. Quantitative PCR reactions were performed on a CFX96 Real Time system (Bio-Rad) with PCR cycle as follows: 95 °C for 10 min, 40 cycles of 95 °C for 10 s and 60 °C for 45 s. The intensity of the HEX/FAM channels at the end of the PCR reaction as compared to positive and negative samples of known genotypes was used for genotype scoring. Chi2 tests were used to compare genotype frequencies between the selected lines and the Tiassale-S line at generation G0.

### RNA-seq library preparation and sequencing

At G17, four pools of 30 3–5 days-old adult females from each line (non blood-fed and not previously exposed to any insecticide) were used for total RNA extraction using Trizol (Life Technologies) according to the manufacturer's instructions. Total RNA was then treated with DNase to remove genomic DNA contaminants. Then, 150 ng of total RNA was used to prepare RNA-seq libraries using the NEBNext® Ultra™ II Directional RNA Library Preparation Kit for Illumina (New England Biolabs) following the manufacturer's instructions. Libraries were quantified using the Qubit DNA BR assay and quality checked on a bioanalyzer (Agilent). Libraries were sequenced in multiplex as 75 bp single reads on a NextSeq 500 (Illumina) by Helixio (Clermont-Ferrand, France). Sequencing output was adjusted to reach > 50 M reads per sample (mean = 57.9 M). After unplexing and quality control using FastQC, the reads were loaded into Strand NGS V 3.2 (Strand Life Sciences) and mapped to the AgamP4 assembly and AgamP4.12 gene set using standard parameters (minimum identity 90%, maximum deviations 5%, minimum aligned length 35 bp, ignore reads with more than 5 matches, cut 3' ends of reads with average quality ≤ 20). After mapping, reads were filtered according to their sequence quality and mapping quality as follows: average read quality ≥ 20, number of N ≤ 5, alignment score ≥ 90, mapping quality ≥ 120, number of matches = 1. Reads passing these quality filters (~ 90% of sequenced reads) were retained for further analyses.

### Differential gene transcription analysis

Differential transcription analysis was performed on all protein-coding genes, with normalization and quantification based on the DE-Seq algorithm [32]. Only genes with a coverage ≥ 4 reads/kb in all replicates across all conditions were retained. Gene mean transcription levels were then compared between each selected line and the parental Tiassalé-S line across all biological replicates using an ANOVA followed by a Tukey HSD test. P-values were adjusted for multiple testing correction using the Benjamini–Hochberg method [33]. Genes with a transcription ratio ≥ 1.5-fold in either direction and a P value ≤ 0.005 in any selected line as compared to the Tiassalé-S line were considered differentially transcribed following insecticide selection. Genes over- and under-transcribed in each selected line were subjected to a Gene Ontology term (GO-term) enrichment analysis using the functional annotation tool DAVID [34]. Reference gene list consisted in all genes detected by RNA-seq. For each line, over- and under-expressed genes were considered separately and GO-terms showing a Fisher’s Exact test P value < 0.05 were considered enriched as compared to the reference list. Heat maps reflecting the transcript profiles of the 126 candidate genes differentially transcribed in any line (i.e. detoxification enzymes including cytochromes P450, carboxy/cholinesterases and transferases; ABC transporters; cuticle proteins; redox enzymes and nervous receptors) were generated using the TM4 Multi-experiment Viewer (Mev) software.

### Polymorphism calling and genome scans

Polymorphisms within transcripts were called using strand NGS V 3.2 on all protein-coding genes of the AgamP4 assembly using default parameters (ignore homopolymeric stretches greater than 4 bp and adjacent positions, coverage ≥ 30 and ≤ 5000, reads supporting the variant allele ≥ 2, base quality ≥ 20, variant confidence score ≥ 200 and strand bias ≤ 25). Of the variations called, only substitutions and indels were retained for further analysis. The following gene effects were then inferred based on AgamP4.12 annotations: synonymous coding, non-synonymous coding, start-lost, stop gained, stop-lost, frameshift coding, splice site, essential splice site, 5′ UTR, 3′ UTR, upstream (within 1500 bp of gene start), downstream (within 1500 bp of gene stop), near gene (within 100 bp of gene).

Selection signatures associated with insecticide selection were investigated using all bi-allelic SNPs (substitutions or indels) that were polymorphic (i.e. showing ≥ 5% allele frequency variation between the Tiassalé-S line and any selected line). A first approach consisted in comparing the mean allele frequency of each SNP between each selected line and the Tiassalé-S line across the four replicates using a Student’s test followed by a Benjamini and Hochberg multiple testing correction [33]. SNPs showing a mean frequency variation between any selected line and the Tiassalé-S line ≥ 50% in either direction and a corrected P value ≤ 0.001 were considered as associated with insecticide selection (Differential SNPs). A second approach consisted in assessing F_ST_ departure from neutrality using the Bayesian method implemented in BayeScan version 2.1 [35]. A separated analysis was performed for each selected line consisting in contrasting the selected line versus the Tiassalé-S line across all replicates. Default settings were used except that prior odd was set to 1000 in order to increase stringency. SNPs showing a Bayscan Q‐value of zero were considered as ‘Outliers’. For each selected line, the proportions of ‘Differential SNPs’ and ‘Outlier SNPs’ per gene were computed and plotted along chromosomes using gene centers as genomic coordinates.

## Results

### Impact of selection on insecticide resistance levels

**Fig. 1:**
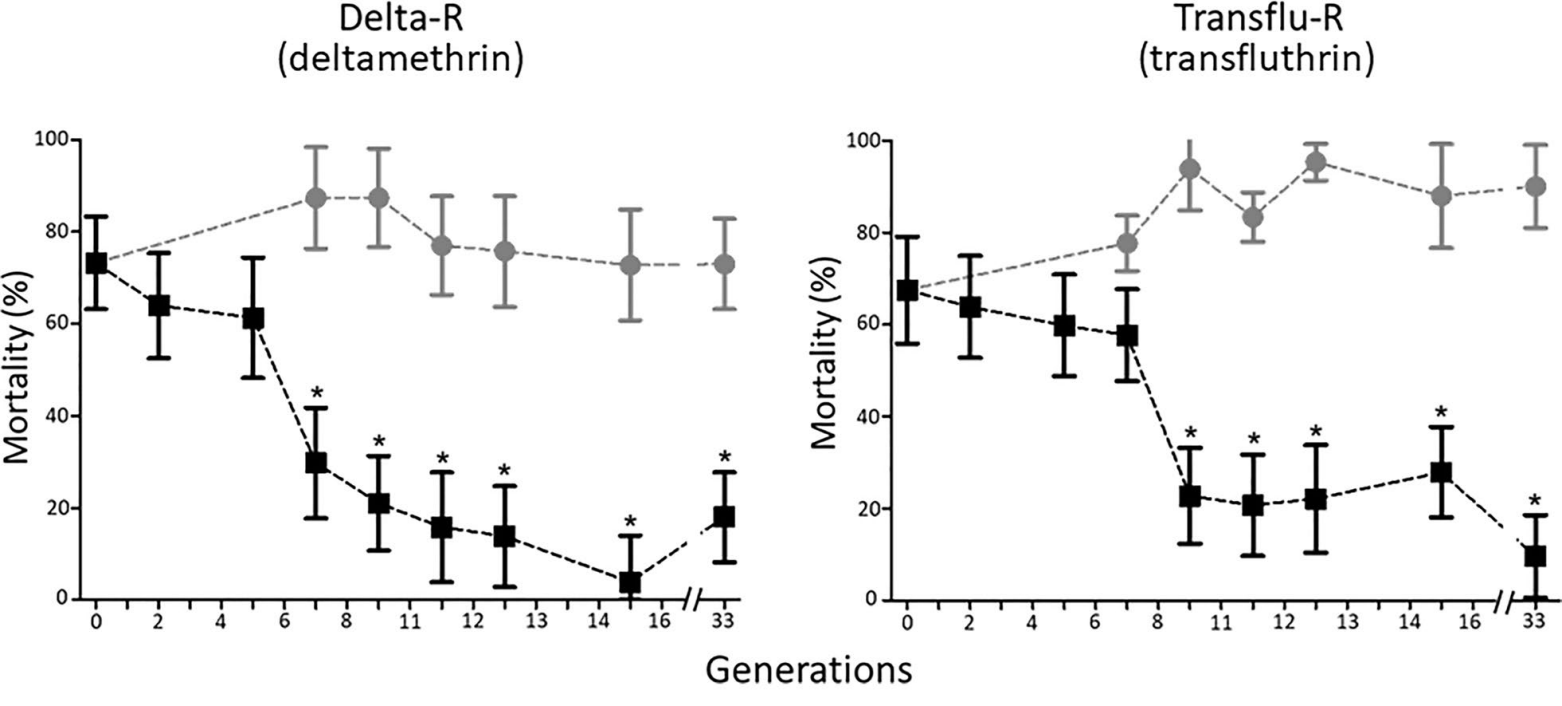
Resistance dynamics of each line along the selection process. Black squares: resistance level of each selected line to the insecticide used for selection. Grey dots: resistance level of the non-selected Tiassalé-S line. Resistance levels were compared using bottle assays and are expressed as mean % mortality ± 95% Wald confidence intervals (N = 5). Comparisons between each line and the non-selected Tiassalé-S line at each generation were performed using a Fisher test on mortality proportions (*p < 0.05)

**Fig. 2:**
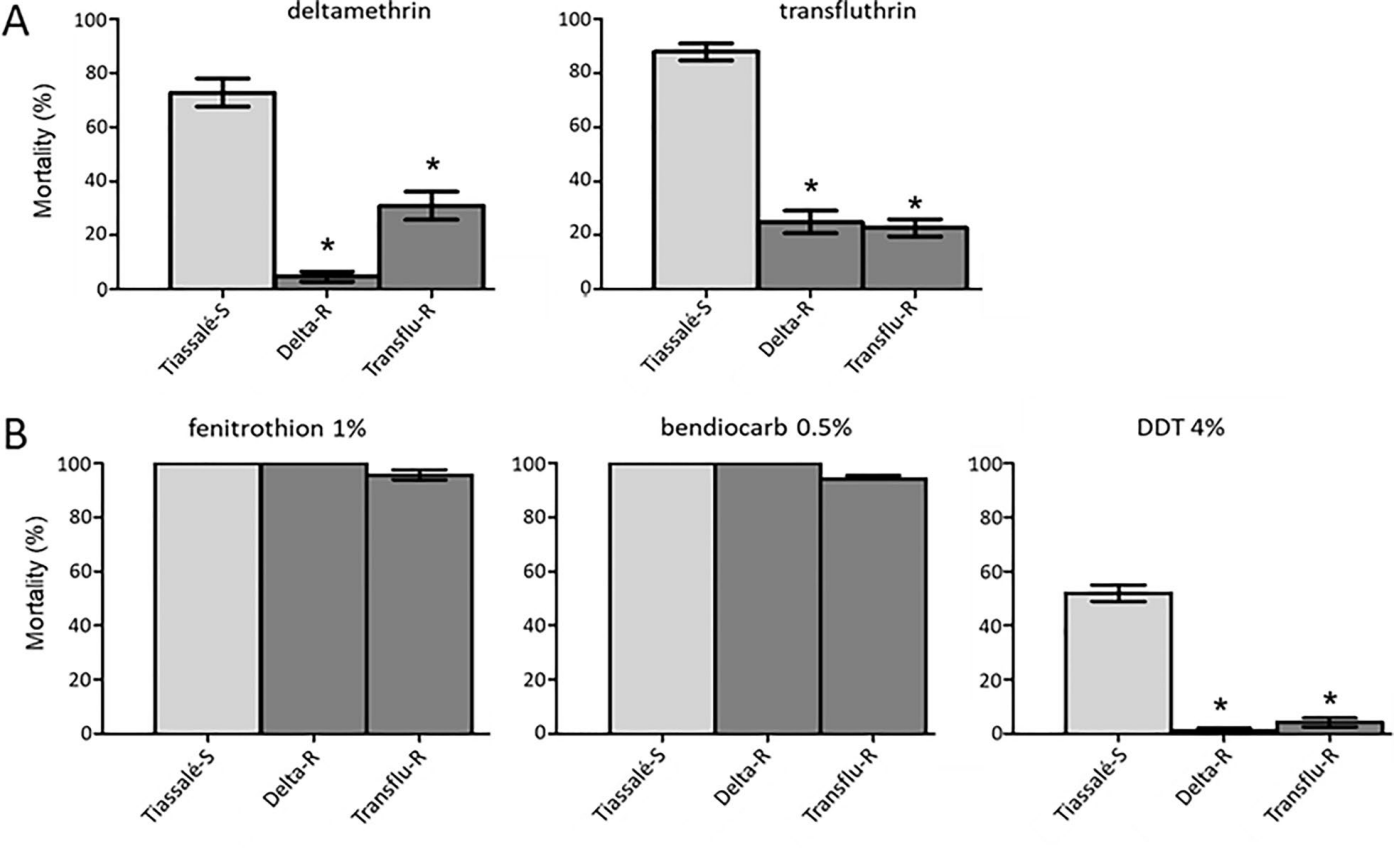
Cross-resistance profiles of each line to insecticides used for selection **A** and to other insecticide families used for vector control **B**. All lines were compared at generation G17. Deltamethrin and transfluthrin resistance levels were compared using bottle assays while resistance levels to other insecticides were compared using WHO test tubes assays. Mortality rates are expressed as mean % mortality ± 95% Wald confidence interval and were compared to the non-selected Tiassale-S line using a Fisher test on mortality proportions (*p < 0.05)

Bioassays confirmed the low resistance level of the Tiassalé-S line at G0 to most insecticide families. Mortality rates were above 95% for deltamethrin, bendiocarb and fenitrothion (Additional file1). However, a significant DDT resistance was observed (46% mortality to 4% DDT). Monitoring resistance levels throughout the selection process showed a gradual increase in deltamethrin and transfluthrin resistance in the Delta-R and Transflu-R lines respectively with (> 55% decreased mortality at G33, P < 0.05 from G7 to G33, Fig. 1). Evaluation of cross-resistance between deltamethrin and transfluthrin at G17 showed a significant cross-resistance of the Delta-R line to transfluthrin and of the Transflu-R line to deltamethrin (P < 0.05, Fig. 2A). Both selected lines also showed a significant increase in DDT resistance with a mortality rate below 10% (P < 0.05). No significant resistance to fenitrothion or bendiocarb was observed in any of the selected lines (P > 0.05, Fig. 2B).

### Impact of selection on the L1014F *kdr* mutation

**Fig. 3:**
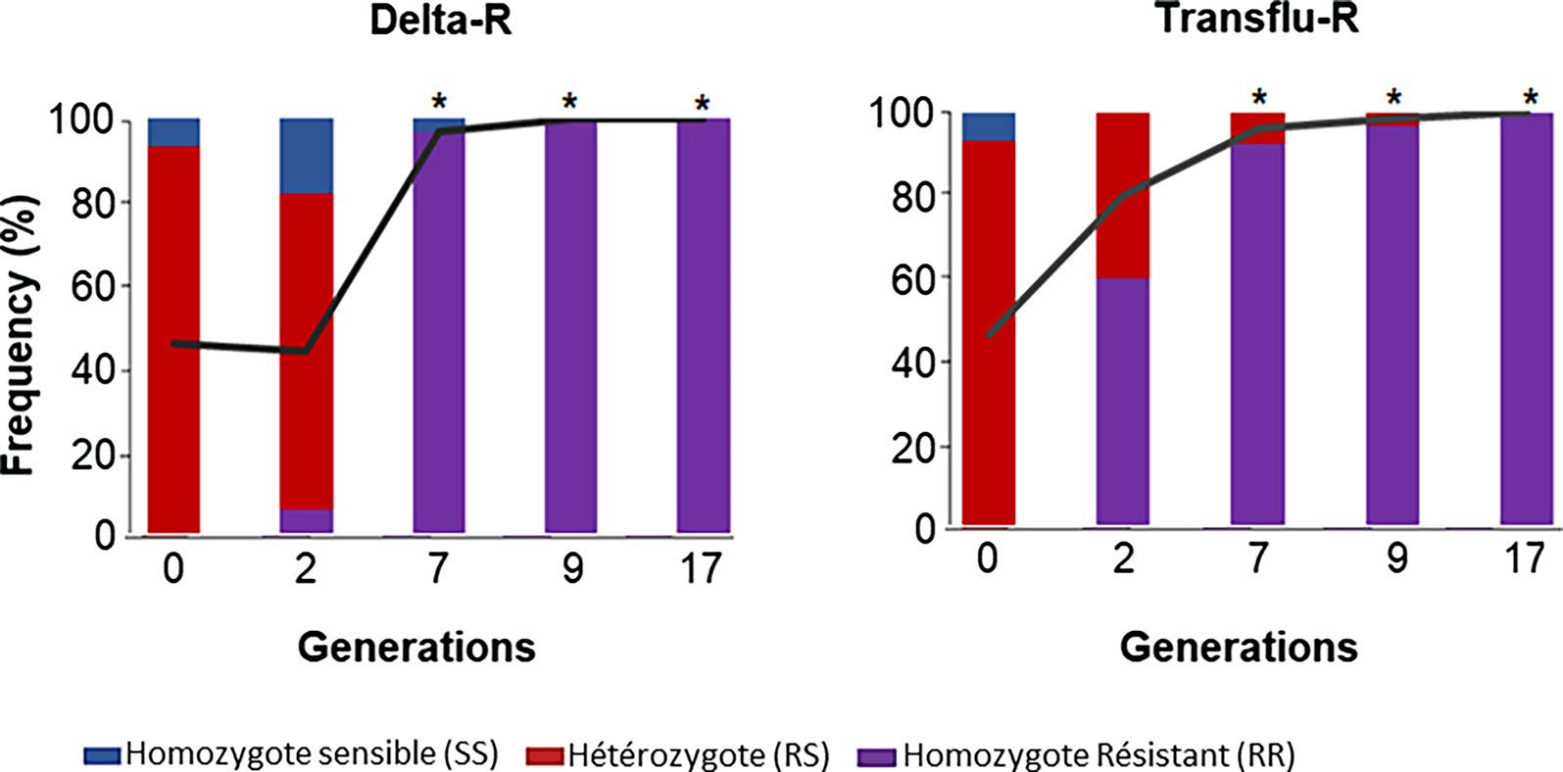
Evolution of L1014F mutation frequency along the selection process. For each line the L1014F *kdr* mutation was genotyped in 30 individual mosquitoes. Genotype frequencies are indicated as pileup charts (purple: f[RR], red: f[RS], blue: f[SS]) and the mean population frequency of the resistant allele is shown as a black line. *indicate a significant genotype frequency difference as compared to the Tiassale-S line at G0 (Chi2 test, P < 0.05)

As expected, after crossing the highly resistant line Tiassalé with the fully susceptible line Kisumu, the Tiassalé-S line carried the *kdr* west mutation (*L1014F*) at a moderate frequency at the beginning of the selection experiment (47%). The frequency of this mutation was significantly impacted by both deltamethrin and transfluthrin selection (Fig. 3). In the Delta-R line, L1014F frequency was estimated to be 98% after 7 generations of selection and reached fixation at generation G9. A similar trend was observed in the Transflu-R line, with an estimated frequency of 92% at generation G9 though fixation was not observed until generation G17.

### Impact of selection on gene transcription levels

RNA-seq allowed detecting 10,829 genes with sufficient read coverage (Additional file 2). Among them, 3174 were differentially transcribed in at least one selected line as compared to the Tiassale-S line (FC > 1.5 in either direction and adjusted P value < 0.005) (Additional file 3). Transfluthrin selection had a broader impact on gene transcription than deltamethrin selection with 1439 and 2786 differentially transcribed genes in the Delta-R and Transflu-R lines respectively. More than 860 genes were specifically over-transcribed in the Transflu-R strain, while only 244 genes were specifically over-transcribed in the Delta-R line as compared to the control line. Nevertheless, 646 genes were simultaneously over-transcribed in both selected lines.

GO terms enrichment analysis revealed a significant enrichment of various biological functions within genes over-transcribed in the Transflu-R line (Additional file 4A). Many enriched functions were related to P450 activity, including 'oxidoreductase activity', 'monooxygenase activity', 'heme binding' and 'iron binding'. An enrichment of the term ‘chitin binding’ associated with cuticle was also observed as well as various terms associated with transcription regulation such as ‘transcription factor activity’ or ‘sequence specific DNA-binding’. In contrast, fewer biological functions were enriched from genes over-transcribed in the Delta-R line, most of them being associated with ion channels and transcription regulation. Terms associated with acetylcholine and G-proteins-coupled receptors were enriched in both selected lines. No biological function that could be associated with known resistance mechanisms were significantly enriched among under-transcribed genes in any selected line, most of them being associated with DNA replication/repair or transcription (Additional file 4B.).

**Fig. 4:**
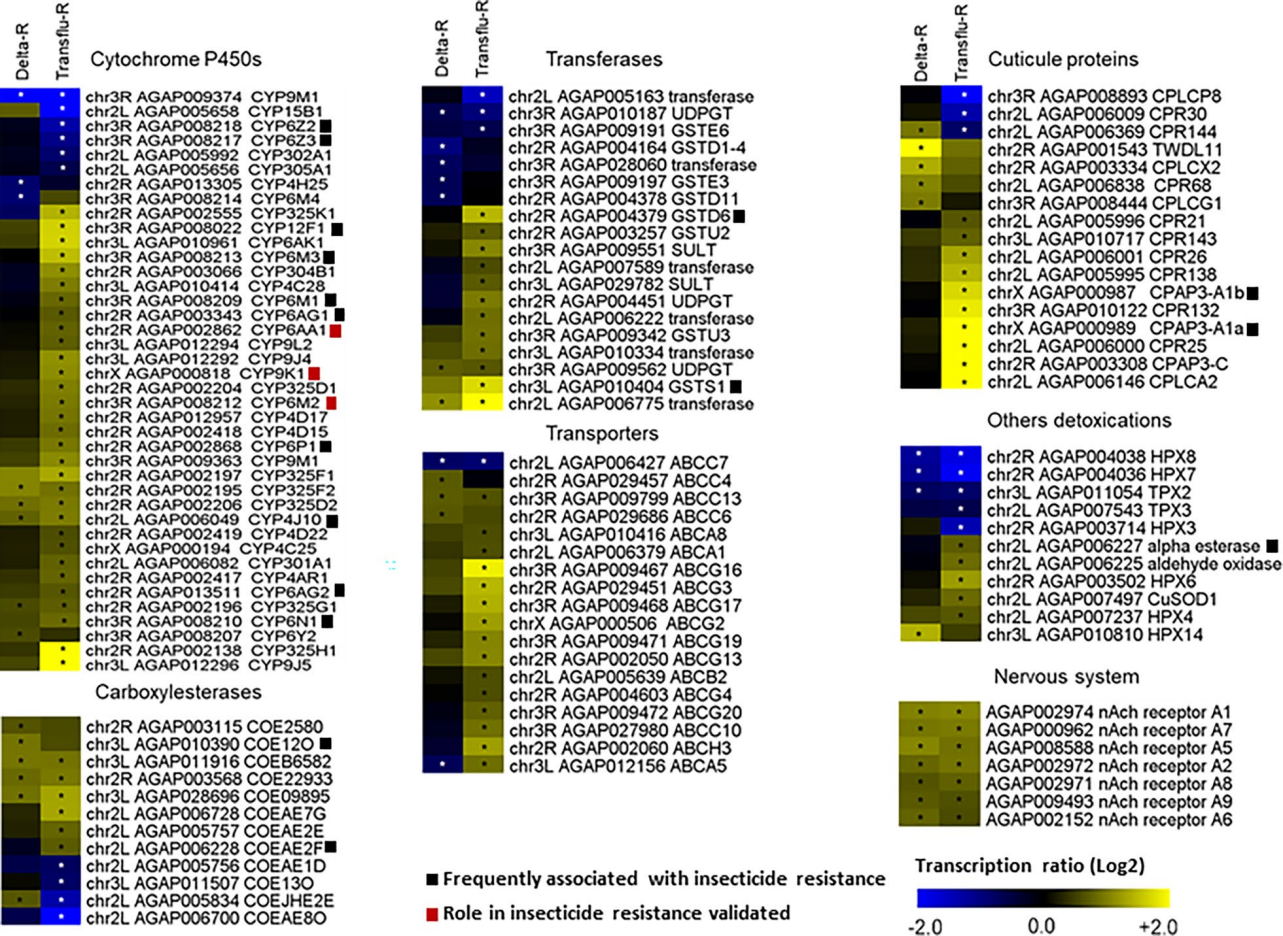
Transcription profiles of candidate genes associated with resistance in each line. All genes significantly differentially transcribed in at least one selected line are shown. Color scale shows the mean Log_2_ Fold Change between each resistant line and the non-selected Tiassale-S line. Stars indicate a significant differential transcription (FC > 1.5-fold and adjusted P value < 0.001)

When considering the gene families classically associated with insecticide resistance, a much larger array of over-transcribed genes was found in the Transflu-R line than in the Delta-R line (Additional file 3). In the Delta-R line, only 29 candidate genes were significantly over-transcribed, including five P450s, six esterases, two transferases, three ABC transporters and five cuticle proteins (Fig. 4 and Additional file 2). Only *CYP4J10* (1.7-fold), have been previously associated with pyrethroid resistance in *An. gambiae* [36]. In contrast, in the Transflu-R line, a total of 88 candidate genes were significantly over-transcribed, including 32 P450s, six esterases, 12 transferases, 15 ABC transporters and 10 cuticle proteins. Within this list, three P450s have been shown to contribute to pyrethroid metabolism in *An. gambiae*: *CYP6AA1* (1.7-fold), *CYP9K1* (2.2-fold) and *CYP6M2* (1.9-fold) [25, 37, 38] while other detoxification or cuticle genes were previously associated with pyrethroid resistance such as *COEAE2F* (1.7-fold), *GSTD6* (2.7-fold) and *GSTS1* (3.3-fold), *CPAP3-A1a* (4.0-fold) and *CPAP3-A1b* (3.3-fold) [39–41]. Eighteen candidate genes were over-transcribed in the two selected lines: four *cyp* genes including *CYP4J10*, three carboxylesterases, two transferases, one ABC transporter and eight genes coding for subunits of the nicotinic acetylcholine receptor.

### Polymorphism variations

**Fig. 5:**
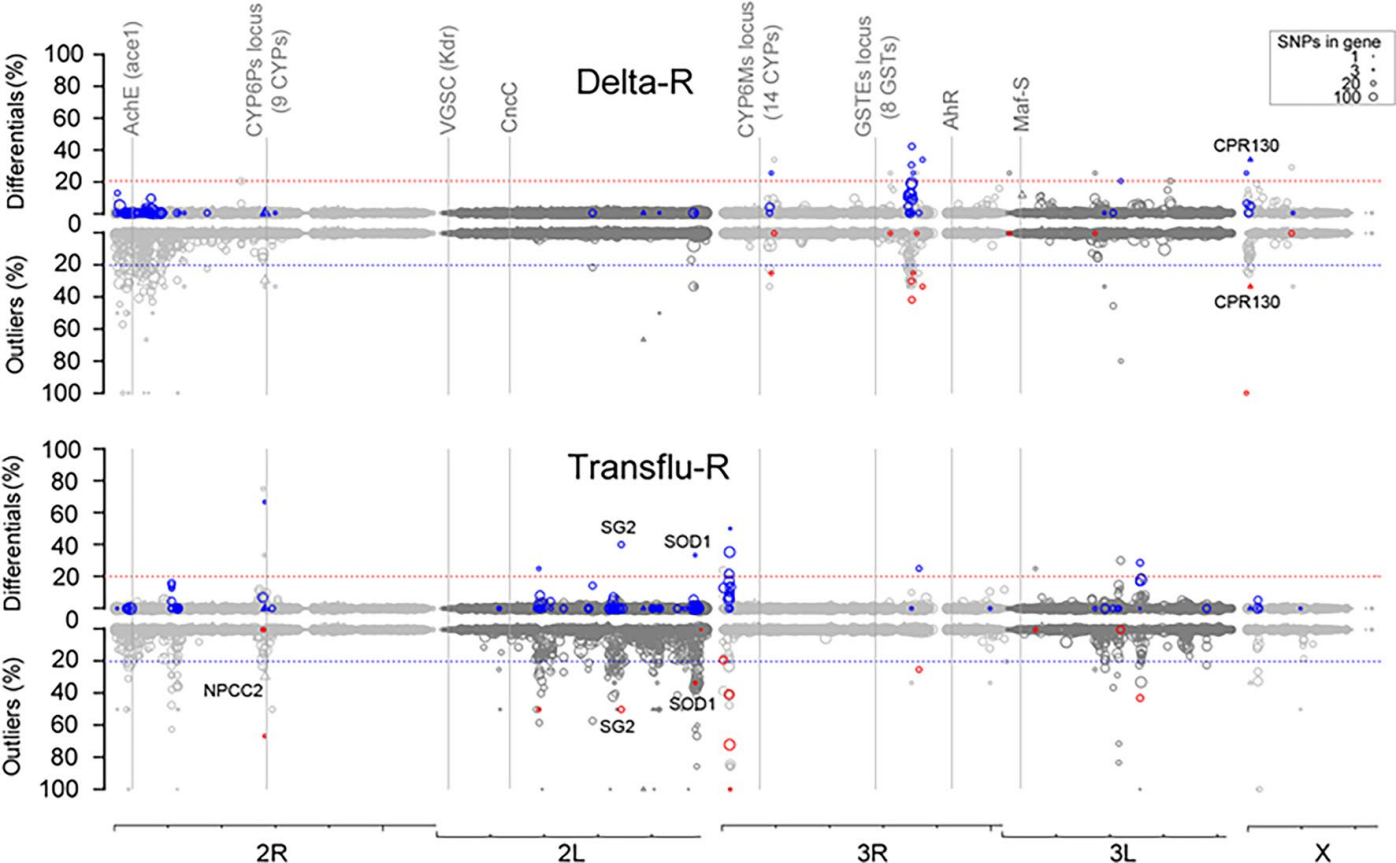
Selection signatures associated with deltamethrin and transfluthrin selection. Selection signatures as compared to the non-selected Tiassalé-S line were inferred using bi-allelic polymorphic SNPs detected by RNA-seq. The upper Y axis shows the proportion of differential SNPs per gene as obtained by the frequency-based approach while the lower Y axis shows the proportion of outliers per gene as obtained by the Fst-based approach. For each approach, blue/red marks indicate genes showing a differential/outlier proportion higher than 20% with the alternative approach (red/blue dashed lines). Symbol size is proportional to the number of polymorphic SNPs per gene. Triangles indicate candidate genes. Loci commonly associated with insecticide resistance in *An. gambiae* are indicated as vertical grey lines. Candidate genes neighbouring major selection signatures are shown. The genomic scale shows chromosome arms with ticks every 10 Mb

Polymorphism data from RNA-seq revealed ~ 130 k biallelic polymorphic SNPs affecting 5574 genes. Of these SNPs, ~ 320 were considered as differentials in each selected line, whereas the less stringent Fst-based method identified 1404 and 2773 outliers SNPs upon deltamethrin and transfluthrin selection respectively (Additional file 5). A total of 70 and 115 genes were affected by both differential and outliers SNPs in the Delta-R and the Transflu-R lines respectively. Only 3 genes (0 candidates) were shared between both selected lines supporting a different response to deltamethrin and transfluthrin selection together with limited drift effects in the control line (Fig. 5).

In the Delta-R line, three specific selection signatures were detected on Chr 3R at ~ 9.18 Mb and ~ 35.3 Mb though no candidate genes were affected or in proximity (± 100 Kb). Another specific selection signature was detected on ChrX at ~ 0.75 Mb centered on the cuticle protein CPR130 and impacting nearby genes.

In contrast, multiple selection signatures were detected in the Transflu-R line, most of them being specific. A strong selection signature was observed on Chr 2R at ~ 28.2 Mb, 97 Kb away from the gene AGAP002848 encoding a Niemann-Pick Type C2 protein (NPC2, a cholesterol-like transporter potentially binding xenobiotics) affected by multiple outliers SNPs. One should note that the *CYP6P* locus previously associated with pyrethroid resistance was located only ~ 250 Kb downstream. This locus contains nine P450s from which two (*CYP6AA1* and *CYP6P3*) were shown to metabolize pyrethroids [22, 37]. Though none of the four *cyp* carrying polymorphic SNPs showed sign of selection within their transcript sequence, two of them (*CYP6AA1* and *CYP6P1*) were specifically over-transcribed in the Transflu-R line. Another selection signature was detected on Chr 2L at ~ 33.1 Mb spanning a cluster of three salivary gland proteins from which *SG2* carried both differential and outlier SNPs. Another specific selection signature was detected downstream at ~ 46.9 affecting the superoxide dismutase *SOD1*. Other specific selection signatures were detected on Chr 3R at ~ 13.9 Mb and on Chr 3L at ~ 24.7 Mb though they were not associated with any candidate gene.

The *vgsc* gene was expressed at a low level in whole adult females for polymorphic SNP to be detected, and as a consequence, no selection signature could be detected at or near the *kdr* locus (Chr 2L at ~ 2.3 Mb). The closest neighbouring genes carrying polymorphic SNPs (located 390 kb upstream and 185 Kb downstream) did not show any sign of selection either. Finally, no major selection signature was detected in any selected line within genes for transcription factors known to affect the expression of detoxification genes such as Cap’n’ Collar (CncC/Nrf2, Chr 2L at ~ 13.8 Mb), the aryl hydrocarbon receptor (AhR, Chr3R at ~ 42.1 Mb) and Maf-S (Chr 3L at ~ 2.8 Mb).

## Discussion

The frequent use of insecticides in agriculture and public health has been shown to promote the evolution of resistance in malaria vectors, which can reduce the effectiveness of vector control measures and may even reverse the progress already made against malaria [18]. This rapid evolution of resistance is under the control of various mechanisms, many of which are not yet fully characterized. Therefore, while there is an urgent need to develop new molecules or combinations of molecules that can replace existing insecticides, it is also important to understand and anticipate resistance and cross-resistance mechanisms in order to maintain their long-term effectiveness in the field. In this concern, the main objective of the study was to perform a multigenerational selection experiment to compare the potential of the polyfluorinated pyrethroid transfluthrin and the phenoxybenzyl pyrethroid deltamethrin to select for resistance in the mosquito *An. gambiae* and to investigate the associated mechanisms using RNA-seq.

The high pyrethroid resistance previously described in the natural population of *An. gambiae* from Tiassalé, Côte d’Ivoire [42, 43] was associated with a high frequency of the *L1014F* mutation (~ 80%). In this study, bioassays performed on the parental line Tiassalé-S, (obtained from a controlled cross between the Tiassalé line and the fully susceptible Kisumu line) confirmed its lower pyrethroid resistance level in association with a lower frequency of the L1014F *kdr* mutation (46%). A few generations of adult selection with deltamethrin or transfluthrin rapidly led to increased resistance to both pyrethroids but also to DDT. Repeated exposure of mosquitoes to an insecticidal compound generally selects for physiological adaptation leading to resistance [40, 44]. As observed in this study, cross-resistance to DDT and pyrethroids has been previously described in *An. gambiae* populations carrying the L1014F/S *kdr* mutations [45]. As expected, monitoring the frequency of the L1014F mutation in the selected lines revealed its concomitant increase in both Delta-R and transflu-R lines, confirming its role in resistance to both pyrethroids. However, although *kdr* mutations have been widely associated with pyrethroid resistance in *An. gambiae*, the key role of other mechanisms such as detoxification, and cuticle alteration has also been demonstrated [40, 41].

RNA-seq revealed the over-transcription of a limited number of detoxification enzymes in the Delta-R line in association with a limited number of selection signatures. The few over-transcribed genes detected did not include detoxification genes known to be involved in pyrethroid metabolism or frequently over-transcribed in pyrethroid resistant mosquitoes, with the exception of *CYP4J10*. Considering the presence of key metabolic resistance alleles such as *CYP6Ms* and *CYP6Ps* in the original Tiassalé population [24], their absence in the Delta-R line may have resulted from the preferred selection of the L1014F *kdr* mutation under high selection pressure. This low metabolic response to deltamethrin selection was confirmed by the absence of enrichment of GO terms associated with detoxification, as well as by polymorphism data showing no selection signatures near key metabolic resistance loci (*i.e. CYP6Ps*, *CYP6Ms*, *GSTEs* gene clusters). In contrast, multiple detoxification genes were over-transcribed in the Transflu-R line in response to transfluthrin selection. This strong metabolic response was supported by an enrichment of GO terms associated with P450s together with a selection signature in the vicinity of a P450 locus previously associated with resistance. Of the over-transcribed P450s, thirteen were previously associated with insecticide resistance in Anopheles. Among them, *CYP6M2*, *CYP9K1*, *CYP6AA1* and *CYP6P1* have been directly linked to pyrethroid resistance in natural populations [46, 47]. For example, a longitudinal study conducted in Benin in 2016 showed an increase in *CYP6M2* expression in *An. gambiae* populations after a mass distribution of long-lasting insecticide-treated nets [41]. *CYP6AA1* was also shown to be over-transcribed in multiple resistant *An. gambiae* populations in Africa [48, 49], while a significant link between increased deltamethrin metabolism and the overexpression of *CYP9K1* was evidenced [50]. In addition, in vitro studies confirmed that *CYP6M2*, *CYP9K1* and *CYP6AA1* can metabolize different types of pyrethroids, including deltamethrin and permethrin [22, 25, 37, 38].

Previous studies combining the use of P450 inhibitors, bioassays and enzymatic assays suggested that the tetrafluorobenzyl ring of transfluthrin makes it less prone to be hydroxylated by P450s in *An. funestus* [26, 27]. However, P450-mediated metabolism of type I and type II pyrethroids may also occur outside of the benzyl ring as shown with *An. gambiae CYP6M2* against deltamethrin [25]. Regarding polyfluorinated pyrethroids, studies conducted in *Culex quinquefasciatus* and *Aedes albopictus* showed a high synergistic effect of the P450 inhibitor PBO on transfluthrin toxicity supporting the capacity of some mosquito P450s to metabolize this pyrethroid [4, 51]. More recently, an in vitro study using the two duplicated alleles of *An. funestus* CYP6P9 confirmed their inability to metabolize transfluthrin through the hydroxylation of its benzyl moiety, but showed their capacity to hydroxylate the *gem*-dimethyl group of the acidic moiety at a moderate rate [51]. Altogether, this suggests that although mosquito P450s are not preferentially attacking the tetrafluorobenzyl ring of transfluthrin, they can still contribute to its metabolism through other oxidative reactions. In this concern, the striking over-expression of multiple *An. gambiae* P450s observed in response to transfluthrin selection may reflect the involvement of multiple P450s in such alternative oxidative pathway. Finally, activity assays showed that transfluthrin is far less effective than deltamethrin on the VGSC [27]. Such lower intrinsic activity on the VGSC might also partially explain the dual selection of both L1014F *kdr* mutation and metabolic resistance alleles in the Transflu-R line as opposed to the preferred selection of the *kdr* mutation in the Delta-R line.

Carboxylesterases (COEs) have also been repeatedly associated with pyrethroid resistance in mosquitoes [15, 47] though not particular *COE* has yet been functionally validated in *An. gambiae*. In mammals, metabolism data suggested that carboxylesterases can mediate transfluthrin hydrolysis [52, 53]. Some COEs were found specifically over-transcribed in the Transflu-R line, including *COAE2F* and the alpha esterase *AGAP006227*, previously reported to be associated with pyrethroid resistance in *An. gambiae* [39, 40]. Though the role of *An. gambiae* COEs in transfluthrin metabolism needs to be further explored, their potential to hydrolyse its ester bonds is supported by the COE-mediated hydrolysis of other pyrethroids showing a similar ester bond [54].

Other gene families previously involved in insecticide resistance were significantly affected in response to transfluthrin selection. Among the multiple cuticle proteins specifically over-transcribed in the Transflu-R line, *CPAP3-A1a CPAP3-A1b*, were frequently found over-transcribed in pyrethroid-resistant populations [41, 46, 55]. Multiple GSTs, UDPGTs and ABC transporters were also specifically over-transcribed in the Transflu-R line. All these gene families were previously involved in pyrethroid resistance in Anopheles although only a few were functionally validated. For example, GSTE2 has been shown to metabolize DDT in various mosquito species but also to contribute to pyrethroid metabolism in *An. funestus* [56]. Though this particular gene was not found over-transcribed nor associated with selection signatures in the Transflu-R line, other *GSTs* such as *GSTD6* and *GSTS1* were previously associated with pyrethroid resistance. ABC transporters are frequently involved in xenobiotic clearance in arthropods [19]. Among those specifically over-transcribed in the Transflu-R line, *ABCG3* and *ABCG4* orthologs were also found over-transcribed in pyrethroid-resistant *Aedes aegypti* and DDT-resistant *Anopheles arabiensis* populations [57], while *ABCH3* was associated with clothianidin resistance in *An. gambiae* [28, 58]. Finally, various nicotinic receptor subunits were over-transcribed in the Delta-R and Transflu-R lines. As described in Perrier and collaborators [59], the 1014F *kdr* mutation could affect presynaptic Na + homeostasis and lead to a decrease in intracellular Ca^2+^ concentration which in turn affects synaptic transmission. Therefore, the overexpression of genes encoding nicotinic receptor subunits observed probably reflects compensatory physiological mechanisms allowing the preservation of synaptic transmission rather than mechanisms directly associated with resistance.

Altogether, these results support the adaptive response of *An. gambiae* to transfluthrin through the selection of the L1014F *kdr* mutation together with an over-transcription of metabolic resistance alleles including some P450s previously associated with resistance to type I and type II pyrethroids. However, it should be kept in mind that the west African mosquito line used here may not totally reflect the genetic variability of *An. gambiae* populations across Africa. Also, the selection regimes applied may not totally reflect those occurring in field conditions, especially in terms of the nature and intensity of selection pressures and population sizes. Finally, polymorphism analysis relied on polymorphic loci detected by RNA-seq, meaning that many genes were overlooked due to their low expression level and that large genomic regions were ignored. For all these reasons, further work is required to further validate the association and the functional role of the transfluthrin resistance markers identified.

## Conclusion

Polyfluorinated pyrethroids such as transfluthrin were suggested as a good alternative to phenoxybenzyl pyrethroids for controlling *Anopheles* mosquitoes in high resistance areas. This idea was supported by previous studies suggesting that the tetrafluorobenzyl ring of transfluthrin makes it less prone to be hydroxylated by mosquito P450s [26, 27, 51]. Although the particular structure of polyfluorinated pyrethroids may effectively provide an additional efficacy against pyrethroid-resistant malaria vectors, the present study suggests that *An. gambiae* can still rapidly adapt to transfluthrin through the selection of the L1014F *kdr* mutation together with other resistance mechanisms. Surprisingly, the adaptive response to transfluthrin includes the overexpression of multiple P450s, among which some confer resistance to pyrethroids currently used in vector control. Overall, these findings call for additional studies to precise the role of P450s and other resistance alleles in transfluthrin resistance and its added value for managing pyrethroid resistance in malaria vectors.

### Supplementary Information

**Additional file 1: **Resistance of the parental line Tiassalé-S to insecticides commonly used for vector control. Insecticide susceptibility tests were performed using WHO test tubes equipped with papers impregnated with 0.05% deltamethrin, 0.5% bendiocarb, 1% fenitrothion and 4% DDT. Exposure time was fixed to 1h and mortality was recorded 24h after exposure. Mortality rates are expressed as mean mortality ± 95% Wald confidence interval.

**Additional file 2: **Transcription data obtained for all genes detected by RNA-seq.

**Additional file 3: **Overview of genes differentially transcribed in the two selected lines. The number of genes is indicated for each line. Numbers within brackets refer to candidate genes potentially involved in insecticide resistance.

**Additional file 4: **Overview of GO term enrichment analysis. Functional pathways enrichment analyses were based on genes significantly over-transcribed in each selected line as compared to Tiassalé-S line using DAVID functional annotation tool (modified Fisher's exact test with P < 0.05). Only GO terms from the « biological process » family showing an enrichment associated with a P value <0.05 and a minimum number of 5 genes are shown. Fold-enrichment (x axis), P value (color scale) and class size (dote size) are indicated. Overview of GO term enrichment analysis. Functional pathways enrichment analyses were based on genes significantly under-transcribed in each selected line as compared to Tiassalé-S line using DAVID functional annotation tool (modified Fisher's exact test with P < 0.05). Only GO terms from the « biological process » family showing an enrichment associated with a P value <0.05 and a minimum number of 5 genes are shown. Fold-enrichment (x axis), P value (color scale) and class size (dote size) are indicated.

**Additional file 5: **Gene-level polymorphism data obtained from RNA-seq.

## Data Availability

RNA-seq sequence data reported in this study have been deposited to the European Nucleotide Archive (ENA, http://www.ebi.ac.uk/ena) under the accession number PRJEB44777.
