# Supplementary figures and images for: Deltamethrin and transfluthrin select for distinct transcriptomic responses in the malaria vector Anopheles gambiae
Source: Malar J. 2023 Sep 4;22:256. doi: 10.1186/s12936-023-04673-5 (PMC10476409; doi:10.1186/s12936-023-04673-5)

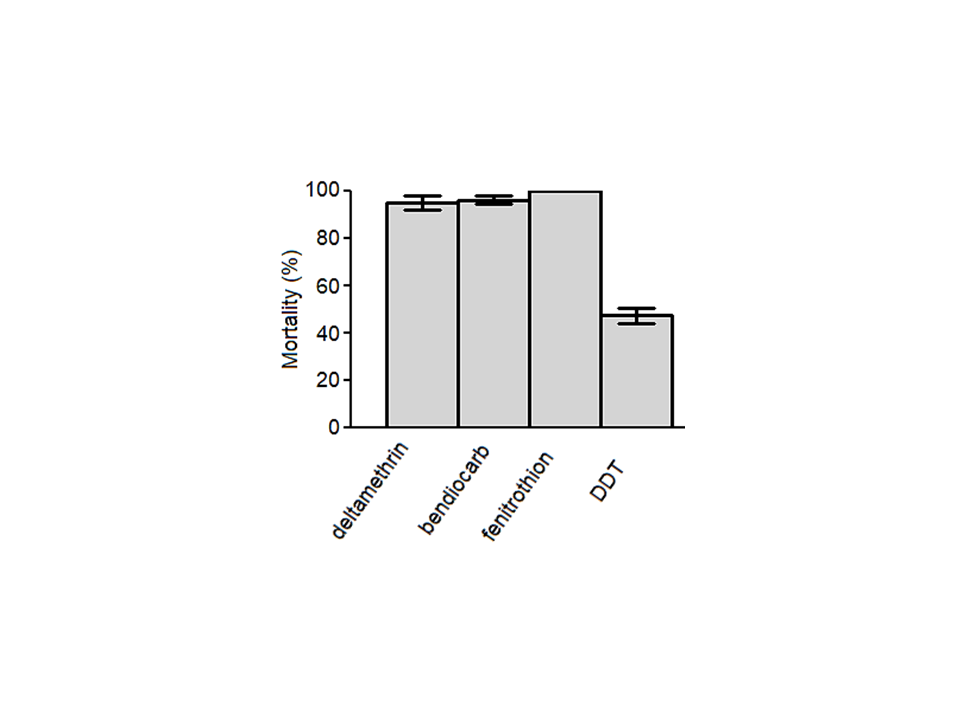

Supplement: Supplementary file 1 — Additional file 1: Resistance of the parental line Tiassalé-S to insecticides commonly used for vector control. Insecticide susceptibility tests were performed using WHO test tubes equipped with papers impregnated with 0.05% deltamethrin, 0.5% bendiocarb, 1% fenitrothion and 4% DDT. Exposure time was fixed to 1h and mortality was recorded 24h after exposure. Mortality rates are expressed as mean mortality ± 95% Wald confidence interval. [file 12936_2023_4673_MOESM1_ESM.tif]

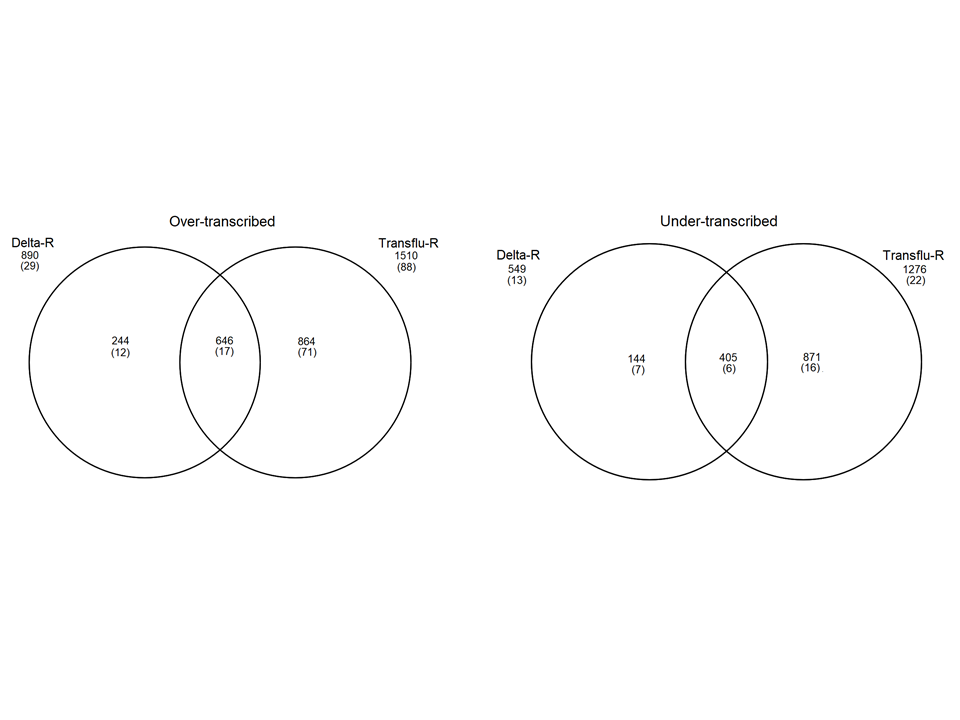

Supplement: Supplementary file 3 — Additional file 3: Overview of genes differentially transcribed in the two selected lines. The number of genes is indicated for each line. Numbers within brackets refer to candidate genes potentially involved in insecticide resistance. [file 12936_2023_4673_MOESM3_ESM.tif]

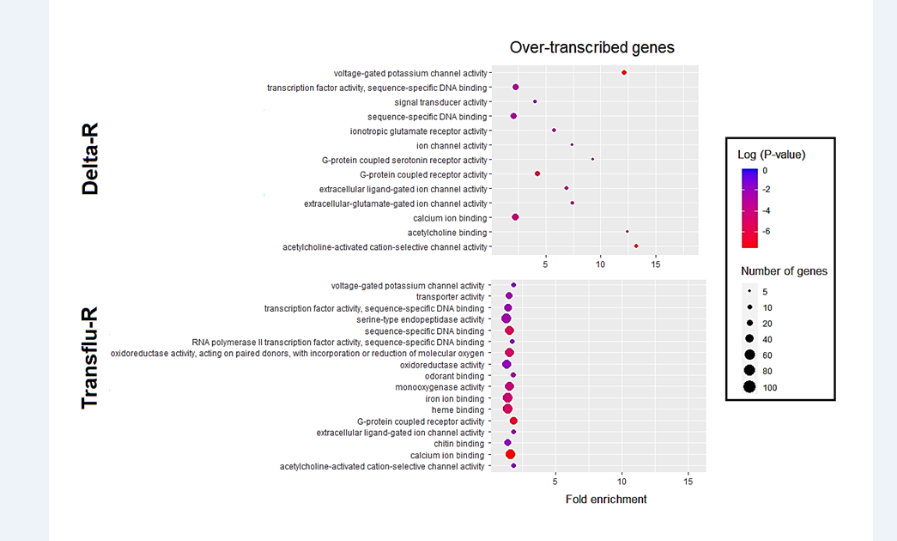


Fig. 4A


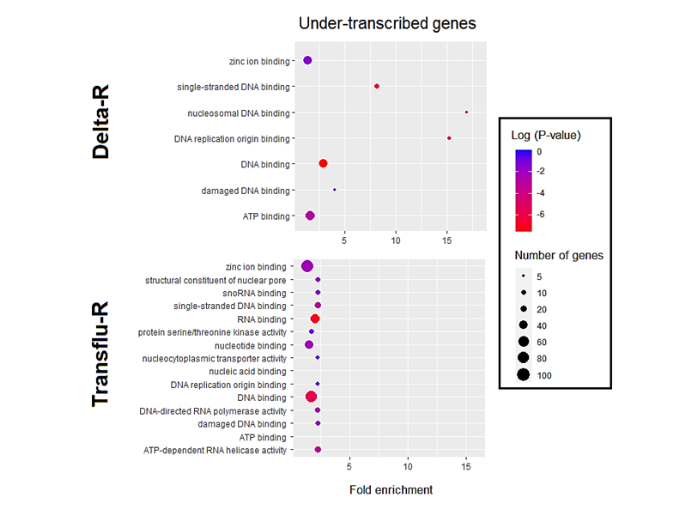


Fig. 4B

Supplement: Supplementary file 4 — Additional file 4: Overview of GO term enrichment analysis. Functional pathways enrichment analyses were based on genes significantly over-transcribed in each selected line as compared to Tiassalé-S line using DAVID functional annotation tool (modified Fisher's exact test with P < 0.05). Only GO terms from the « biological process » family showing an enrichment associated with a P value <0.05 and a minimum number of 5 genes are shown. Fold-enrichment (x axis), P value (color scale) and class size (dote size) are indicated. Overview of GO term enrichment analysis. Functional pathways enrichment analyses were based on genes significantly under-transcribed in each selected line as compared to Tiassalé-S line using DAVID functional annotation tool (modified Fisher's exact test with P < 0.05). Only GO terms from the « biological process » family showing an enrichment associated with a P value <0.05 and a minimum number of 5 genes are shown. Fold-enrichment (x axis), P value (color scale) and class size (dote size) are indicated. [file 12936_2023_4673_MOESM4_ESM.docx]
